# Monitoring Land Subsidence in Wuhan City (China) using the SBAS-InSAR Method with Radarsat-2 Imagery Data

**DOI:** 10.3390/s19030743

**Published:** 2019-02-12

**Authors:** Yang Zhang, Yaolin Liu, Manqi Jin, Ying Jing, Yi Liu, Yanfang Liu, Wei Sun, Junqing Wei, Yiyun Chen

**Affiliations:** 1 School of Resource and Environmental Sciences, Wuhan University, Wuhan 430079, China; zhangy1010@whu.edu.cn (Y.Z.); liuyaolin2010@163.com (Y.L.); kingerin@163.com (M.J.); y.crystal@whu.edu.cn (Y.J.); liuyi2010@whu.edu.cn (Y.L.); weijunqing@whu.edu.cn (J.W.); 2 Key Laboratory of Geographic Information System, Ministry of Education, Wuhan University, Wuhan 430079, China; 3 Collaborative Innovation Center for Geospatial Information Technology, Wuhan 430079, China; 4 Wuhan Geomatics Institute, Wuhan 430022, China; gnss.wei@gmail.com

**Keywords:** land subsidence, Radarsat-2 images, small baseline subset (SBAS) method, interferometric synthetic aperture radar (InSAR)

## Abstract

Wuhan city is the biggest city in central China and has suffered subsidence problems in recent years because of its rapid urban construction. However, longtime and wide range monitoring of land subsidence is lacking. The causes of subsidence also require further study, such as natural conditions and human activities. We use small baseline subset (SBAS) interferometric synthetic aperture radar (InSAR) method and high-resolution RADARSAT-2 images acquired between 2015 and 2018 to derive subsidence. The SBAS-InSAR results are validated by 56 leveling benchmarks where two readings of elevation were recorded. Two natural factors (carbonate rock and soft soils) and three human factors (groundwater exploitation, subway excavation and urban construction) are investigated for their relationships with land subsidence. Results show that four major areas of subsidence are detected and the subsidence rate varies from −51.56 to 27.80 millimeters per year (mm/yr) with an average of −0.03 mm/yr. More than 83.81% of persistent scattered (PS) points obtain a standard deviation of less than −6 mm/yr, and the difference between SBAS-InSAR method and leveling data is less than 5 mm/yr. Thus, we conclude that SBAS-InSAR method with Radarsat-2 data is reliable for longtime monitoring of land subsidence covering a large area in Wuhan city. In addition, land subsidence is caused by a combination of natural conditions and human activities. Natural conditions provide a basis for subsidence and make subsidence possible. Human activities are driving factors and make subsidence happen. Moreover, subsidence information could be used in disaster prevention, urban planning, and hydrological modeling.

## 1. Introduction

Land subsidence is defined as a gradual settling or sudden sinking of the ground surface [1,2,3], which results from natural processes or human activities [4,5,6,7]. Over the past decades, numerous land subsidence events have been reported in many cities around the world where the rapid urban construction and the extensive groundwater exploitation are taking place [8,9,10,11,12,13]. Land subsidence can lead to serious environmental problems and considerable economic losses, such as damage to infrastructures and increased risk of urban pluvial flooding [14,15,16,17,18]. Thus, the demand for monitoring the spatial and temporal distribution of land subsidence is increasing.

Traditional point-based monitoring approaches such as ground leveling and global positioning system (GPS) techniques could not provide sufficient samples required by land subsidence mapping [19]. In recent years, interferometric synthetic aperture radar (InSAR) technology has been rapidly developed to cover a large geographic area. InSAR method is low-cost and effective [20,21]. Nevertheless, the InSAR method suffers from temporal decorrelation and atmospheric disturbance [22,23,24]. Therefore, many advanced InSAR methods based on multi-interferograms such as persistent scatterer interferometry (PS-InSAR) and small baseline subset interferometry (SBAS-InSAR) have been proposed to overcome these limitations [25,26,27,28,29]. Furthermore, high-resolution SAR images are gradually applied such as ALOS-PALSAR and Radarsat-2 images [30,31].

The primary cause of land subsidence is human activities, such as groundwater withdrawal, coal mining, petroleum extraction, land creation, subway excavation, and building loading [4,21,29,32,33,34,35,36]. Besides, natural factors might also be critical, such as soft soil, karst geomorphologic [37,38]. Previous studies have examined the cross-correlations between these factors and land subsidence [39,40]. However, it remains unclear whether human factor works alone or with natural factor. Thus, the roles of natural and human factors in land subsidence require further study.

Wuhan city, which is the biggest city in central China, has various types of natural conditions and has experienced rapid urbanization in recent years. It is a typical city to study the problem of land subsidence in China. Previous studies have mapped land subsidence in Wuhan city using advanced InSAR methods [5,41,42]. However, longtime monitoring of land subsidence covering all urban areas of Wuhan city is lacking. In addition, Radarsat-2 images have not yet been applied to subsidence monitoring in Wuhan city.

This study explores the application of SBAS-InSAR method with high-resolution Radarsat-2 images to long-term monitoring of land subsidence in Wuhan city, and the cause of land subsidence. Specifically, (i) we investigate the potentials of 20 Radarsat-2 images acquired between 17 October 2015 and 3 June 2018 to derive land subsidence rates in Wuhan city. (ii) The InSAR results are validated by 56 leveling benchmarks. (iii) We study the influence of natural conditions and human activities on land subsidence and their interrelationships.

## 2. Study Area and Data Preparation

### 2.1. Study Area

**Figure 1:**
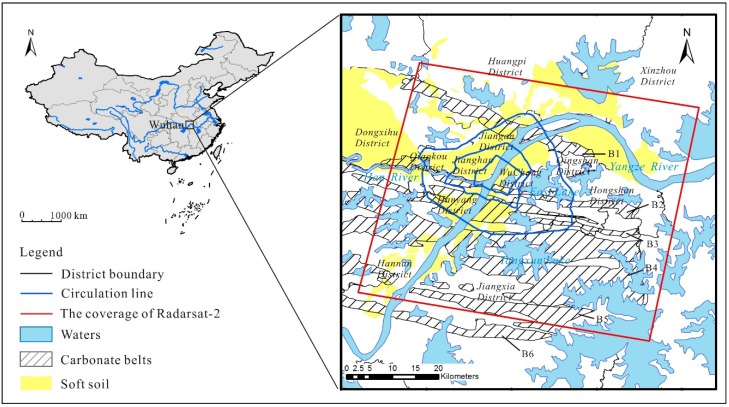
The location of Wuhan city in China and the study area. The red rectangle illustrates the coverage of Radarsat-2. B1–B6 represent six carbonate rock belts aligned in an East-West orientation, namely Tianxingzhou, Daqiao, Baishazhou, Zhuankou, Junshan, and Hannan.

Wuhan city (29°58′ N–31°22′ N, 113°41′ E–115°05′ E) is located in the east of an alluvial plain called Jianghan Plain, see Figure 1. The Yangtze River, the world’s third longest river, flows through the heart of the city. The average elevation of the city is about 37 m. About 26% of total area (2205.06 km^2^) is covered by water [43], such as rivers, lakes, ponds and ditches. The city has a subtropical monsoon climate characterized by four distinct seasons, abundant precipitation, and considerable sunshine. The average annual temperature is 16.6 °C and the precipitation averages 1269 mm. The rainfall concentrates in early summer (May to July) [44].

Carbonate rock and soft soils, which might contribute to land subsidence, are widespread in Wuhan city, see Figure 1. There are six carbonate rock belts aligned in an East-West orientation, and they cover an area of more than 1100 km^2^ [45,46,47]. Soft soils have high water content, high compressibility, high porosity and low shear strength. Soft soils are mainly distributed along the banks of two rivers, the Yangtze River and the Han River, and the maximum thickness exceeds 10 m [48,49]. Wuhan city has experienced rapid economic growth since the China’s reform and opening up policy in 1979. It has become a megacity with a population in excess of 10 million.

### 2.2. Datasets

We employ 20 descending Radarsat-2 wide ultra-fine (WUF) single-look complex (SLC) images acquired from October 2015 to June 2018 at intervals of 24, 48, 72 or 96 days. These single horizontal-horizontal (HH) polarization images covered a 50 × 50 km area, see the red rectangle in Figure 1. Main parameters of Radarsat-2 WUF SLC data are detailed in Table 1. The Shuttle Radar Topography Mission (SRTM) 90 m DEM is used to simulate and remove topographic phases. To validate the InSAR results, we also employ 56 leveling benchmarks where two readings of elevation were recorded in September 2016 and March 2017, respectively.

We gathered data about natural and human factors that influence land subsidence. Two natural factors include soft soil and carbonate rock, see Figure 1. A map of soft soils distribution and a map of carbonate belts distribution are obtained from Wuhan municipal commission of urban-rural development and a geological study, respectively [47]. Three human factors are considered: groundwater exploitation, subway excavation and urban construction. The data of the three human factors include an official route map of the Wuhan subway system, the groundwater resources regionalization of Wuhan, two high resolution images of the year 2015 and 2017. In addition, impervious surface fraction is an index that measures the level of urban construction [50].

## 3. Methodology

**Figure 2:**
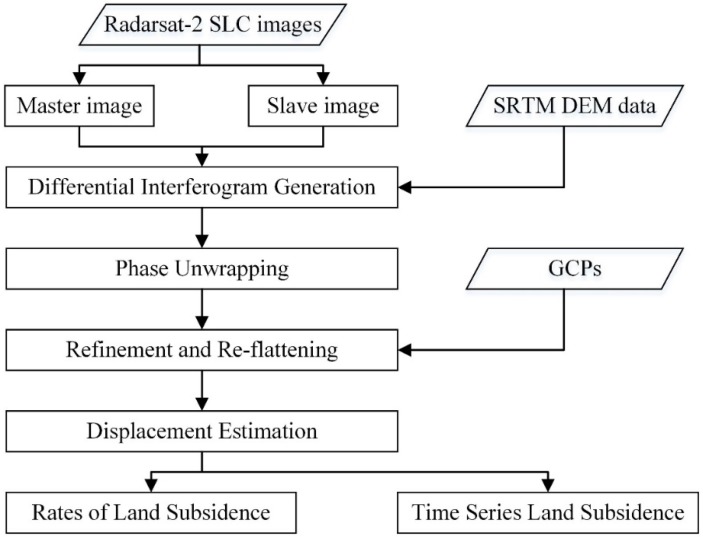
Flowchart of SBAS-InSAR data processing.

The SBAS-InSAR method is used to process Radarsat-2 WUF SLC images in the ENVI SARScape module to obtain land subsidence information in Wuhan city [31]. The SBAS-InSAR method is an advanced InSAR technique that could improve the monitoring accuracy [51]. The SBAS-InSAR method relies on an appropriate combination of differential interferograms within the thresholds of temporal and spatial baselines, so the geometric decorrelation is minimal [26,31,36,52]. Figure 2 shows the main steps of SBAS-InSAR method to detect land subsidence.

### 3.1. Differential Interferogram Generation

**Figure 3:**
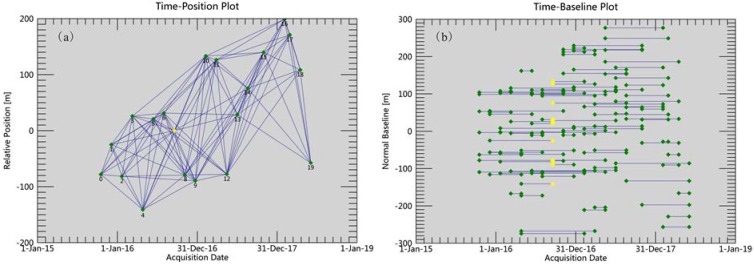
(**a**) Time–position of Radarsat-2 image interferometric pairs and (**b**) time–baseline of Radarsat-2 image interferometric pairs. The yellow diamond denotes the super master image. Blue lines represent interferometric pairs. Green diamonds denote slave images.

The image acquired on 17 September 2016 is selected as the super master image, and the remaining 19 images are slave images. The selection of interferograms is constrained by a maximum spatial baseline of 630 m (45% of the critical spatial baseline) and a maximum temporal baseline of 350 days. After topographic phase removal, 106 differential interferograms are generated, see Figure 3. The signal-to-noise ratio is improved by performing multi-looking factors of 4 × 4 in the range and azimuth directions, and Goldstein filtering method.

### 3.2. Phase Unwrapping

Both minimum cost flow (MCF) network and Delaunay 3D are employed for phase unwrapping, and a coherence threshold of 0.35 is chosen [35]. Then, 39 interferometric pairs with poor unwrapping and low coherence are eliminated.

### 3.3. Refinement and Re-flattening

After phase unwrapping, 46 Ground Control Points (GCPs) are selected to correct the unwrapped phase. The selection criteria are as follows: (1) the location has a high coherence value and good phase unwrapping, (2) land deformation is close to zero according to previous studies and leveling data, and (3) we should select as many GCPs as possible.

### 3.4. Displacement Estimation

Preliminary displacements are estimated by a linear model that is robust and commonly used [36]. Meanwhile, the residual topography is also removed. Then, atmospheric phase was removed by an atmospheric filtering. Subsequently, geocoding in the line of sight (LOS) direction with a resolution of 10 m is employed to calculate SBAS. Finally, subsidence rate and subsidence time series are obtained and mapped across the study area.

### 3.5. InSAR Data Validation by Using Leveling Benchmarks

The InSAR results are validated by 56 leveling benchmarks. Among these leveling benchmarks, a stable one located at East Lake Peony Garden (30°34′27″ N, 114°21′57″ E) is used as a reference point to measure land subsidence. Four parameters, namely, maximum discrepancy (MaxD), minimum discrepancy (MinD), mean absolute discrepancy (MD), and root mean square (RMS), are used to describe the reliability of SBAS-InSAR derived land subsidence rate map.

## 4. Results and Validation

### 4.1. Rates of Land Subsidence

**Figure 4:**
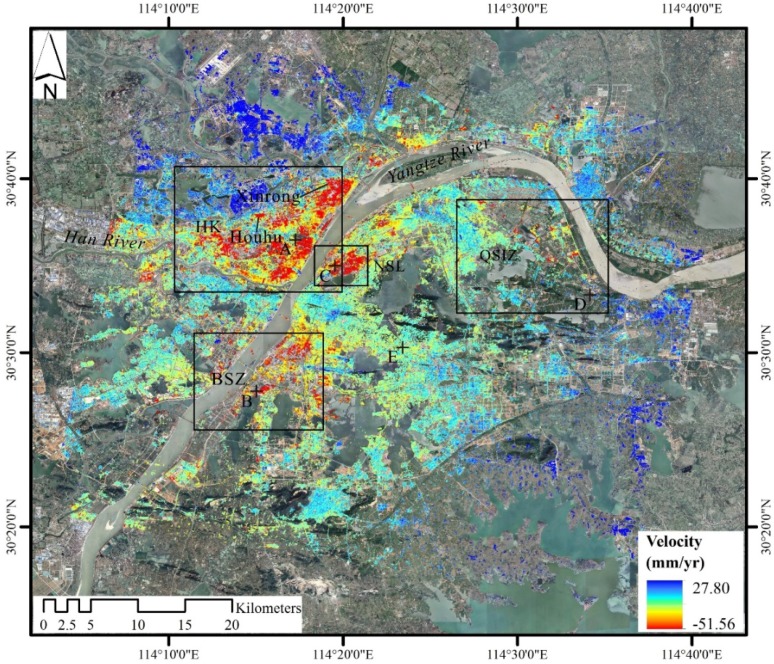
The average subsidence velocity in LOS from October 2015 to June 2018 across Wuhan city by using SBAS-InSAR technique. The four black rectangles are the four major areas of subsidence. A-E are five points of subsidence, detailed in Figure 6.

Figure 4 shows the average subsidence velocity in the radar LOS from October 2015 to June 2018 across Wuhan city by using SBAS-InSAR technique. A negative value (in red color) indicates land subsidence, and a positive value (in blue color) indicates uplift. The total number of derived permanent scatter (PS) points was 8,680,765, and the average density was 3472 points/km^2^. The subsidence rate varies from −51.56 to 27.80 millimeters per year (mm/yr) with an average of −0.03 mm/yr. Additionally, a pronounced subsidence area located in Hankou district, adjacent to the Xinrong Light Rail Transit station with a maximum velocity exceeding −50 mm/yr, is identified.

Land subsidence is widely found in most areas of the city, and land uplift in surrounding rural areas is also apparent (Figure 4). Four major areas of subsidence are detected: Hankou (HK), Qingshan Industrial Zone (QSIZ), Northern Shahu Lake (NSL), and Baishazhou (BSZ). HK covers the largest subsidence area, and is the main commercial district of the city. QSIZ is the city’s oldest and biggest industrial area, and there are many large manufacturing plants, such as Wuhan Iron and Steel (Group) Corporation, Wuhan Petrochemical Complex, and Qingshan Thermal Power Plant. NSL has been undergoing rapid economic growth and high intensity of urban construction over the years. BSZ is located in the south of the city, and has speed up the construction of traffic facilities. Interestingly, all four major areas of subsidence are distributed along the banks of the Yangtze River. Other areas of subsidence are sinking slowly at a rate of less than −10 mm/yr.

### 4.2. Evolution of Land Subsidence

**Figure 5:**
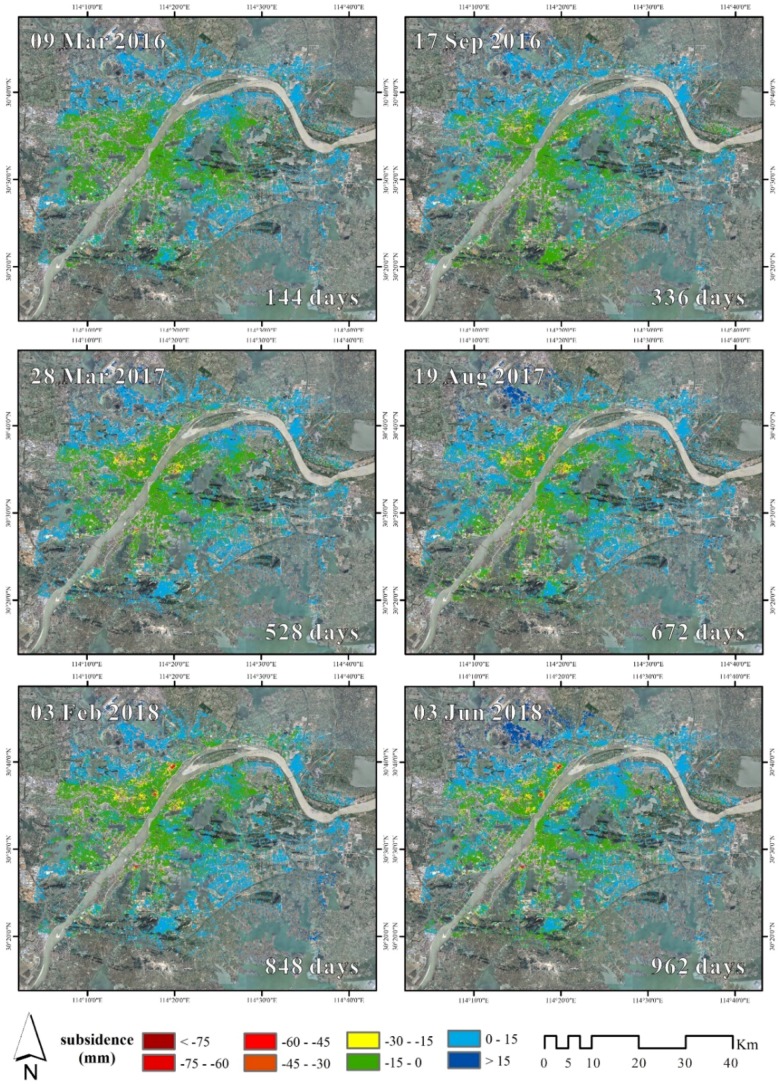
Spatio-temporal evolution of accumulated subsidence in Wuhan city derived from Radarsat-2 images. Only 6 of the 20 subsidence maps are shown.

Figure 5 illustrates the spatial distribution of subsidence and its changes over time. In most part of the city, the cumulative subsidence is stable in a range of −15 to 15 mm. But for the four major areas of subsidence, the cumulative subsidence gradually increases over time, and the area is constantly expanding. The maximum cumulative subsidence has reached up to −126.43 mm, is located in Xinrong of HK, see Figure 4.

**Figure 6:**
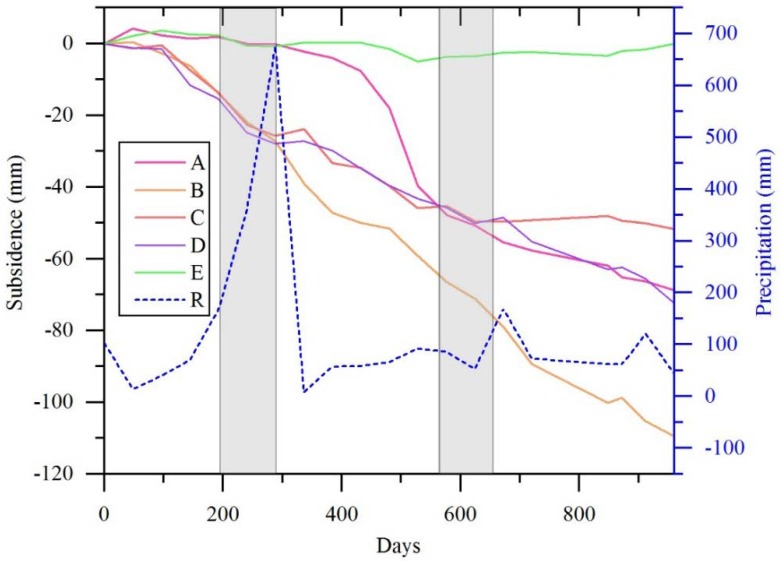
Time-series subsidence at the five typical points A–E. The gray rectangle denotes the early summer (May, June, and July).

The time series of subsidence at five typical PS points marked as A–E in Figure.4, is shown in Figure 6. Points A, B, C, and D are located in HK, BSZ, NSL, and QSIZ, respectively, which are the four major areas of subsidence. Point E is located in an urban area with minor subsidence of nearly zero mm. Points A, B, C, and D present nonlinear subsidence. One possible reason is that the seasonal variation of groundwater levels might influence the rate of subsidence. When in early summer (May, June, and July) rainfall concentrates, groundwater will be recharged and the rate of subsidence will slow down, see Figure 6. Points B, C, and D show similar trends of subsidence, and point B subsides more than points C and D. The subsidence at point A suddenly increases in 2017 probably due to the construction of Wuhan Metro Line No. 8.

### 4.3. InSAR Data Validation

Statistical analysis of the mean standard deviations is conducted to assess the internal precision of subsidence rates of subsidence rates. More than 83.81% of PS points obtain a standard deviation of less than -6 mm/yr, proving that applying SBAS-InSAR method to derive subsidence rates is reliable.

**Figure 7:**
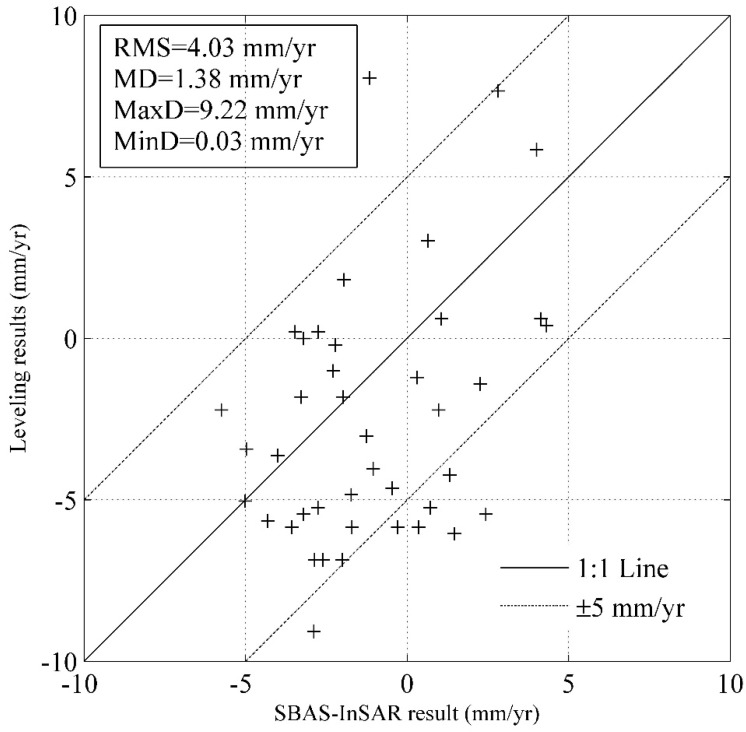
Leveling data versus SBAS-InSAR method plots of land subsidence.

The land subsidence derived from Radarsat-2 images are compared to those derived from leveling data (Figure 7). 41 out of 56 leveling benchmarks are located within the generated grids, and are selected for validation. Figure 7 shows the results of leveling data against SBAS-InSAR method. For most validation points, the difference between the two methods is less than 5 mm/yr. MaxD, MinD, MD, and RMS are 9.22, 0.03, 1.38, and 4.03 mm/year, respectively. The result of SBAS-InSAR coincides with that of leveling data, which indicates that SBAS-InSAR method is able to monitor land subsidence with acceptable precision.

## 5. Discussion

### 5.1. Comparison with Previous Studies

In this study, SBAS-InSAR method with Radarsat-2 data is reliable for longtime monitoring of land subsidence covering a large area in Wuhan city (October 2015 to June 2018). We also compare our results with those of the following studies (Table 2).

Zhou et al. [5] obtained the rate of subsidence in Wuhan city by using SBAS-InSAR method with 15 Sentinel-1A images (April 2015 and April 2016) with 5 m × 20 m (range × azimuth) spatial resolution. Their results showed that subsidence rates varied from−82 mm/yr to 18 mm/yr, and the maximum rate of subsidence was detected in Houhu of HK. In addition, there are several centers of subsidence areas in Wuchang, Qingshan, Hanyang, and Hongshan district.

Bai et al. [41] investigated the rate and spatial patterns of subsidence in major urban areas in Wuhan city using PS-InSAR method with TerraSAR-X images (October 2009 and August 2010) with 2.0 m × 3.3 m (range × azimuth) spatial resolution. Subsidence rates varied from−63.7 mm/yr to 17.5 mm/yr, and HK is the largest subsidence area.

Costantini et al. [42] obtained subsidence information from high-resolution X-band COSMO-SkyMed data (June 2013 to June 2014) with 2.21 m × 1.63 m (range × azimuth) spatial resolution using PS pair InSAR method. Subsidence rates of most PS points in HK varied from −80 mm/yr to 40 mm/yr.

Benattou et al. [53] measured the rate of subsidence using 36 sentinel-1A images (June 2015 and April 2017) with 5 m × 20 m (range × azimuth) spatial resolution. The average deformation ranged from −127 mm/yr to 23 mm/yr and a new center of subsidence areas (Jiufengxiang) was found.

In our study, four major areas of subsidence are clearly identified, namely, HK, QSIZ, NSL, and BSZ, which are consistent with earlier research conducted by Zhou et al. However, the maximum rate of subsidence is −52 mm/yr, which is lower than the maximum rate of −82 mm/yr by Zhou et al. It is also lower than the rate of −67 mm/yr conducted by Bai et al. and −127 mm/yr conducted by Benattou et al. The reason behind this is that subsidence might occur over a short period of time and the rate of longtime monitoring would be relatively lower. Our longtime monitoring of land subsidence reflect a long term change of land subsidence relative to previous studies. The most severe ground settlement site of our study is located at Xinrong of HK, but in the study of Zhou et al. it is located at one other place named Houhu (Figure 4). Compared to the work of Bai et al. some places within major areas of subsidence exhibit a considerable increase in subsidence velocity. For example, the subsidence velocity in NSL is between −15 mm/yr and 5 mm/yr in the study of Bai et al. during 2009–2010, but it exceeds −15 mm/yr in our study during 2015–2018. By comparing and analyzing the results of subsidence monitoring at different times, the law of land subsidence over time in Wuhan city can be revealed.

### 5.2. Causes of Subsidence in Wuhan City

#### 5.2.1. Natural Factors

**Figure 8:**
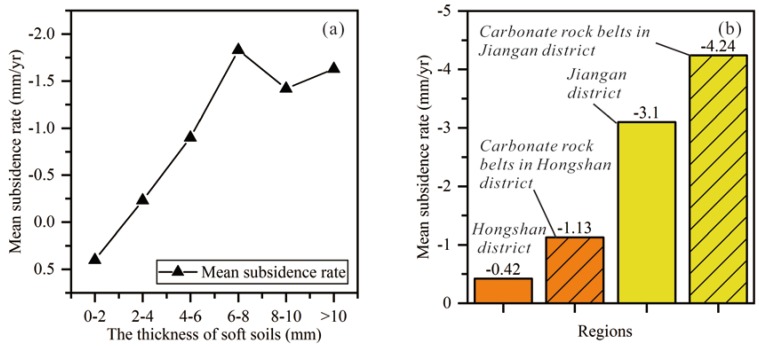
(**a**) Relationship between soft soil thickness and subsidence rate. (**b**) The subsidence rate of areas located on carbonate rock belts and those of the whole of the two urban areas.

In Wuhan city, carbonate rock and soft soils are widespread and might cause land subsidence (Figure 1 and Figure 4). For the four major areas of subsidence, BSZ and QSIZ are located on the carbonate rock belts, and HK and NSL are located on the soft soils. Obviously, there exists a spatial correlation between land subsidence and the two natural factors. The rate of subsidence increases with the thickness of soft soils (Figure 8a). Taking Hongshan district and Jiangan district (Figure 1) as examples, we compare areas located on carbonate rock belts with the whole of the two urban areas (Figure 8b). The subsidence rate of areas on carbonate rock belts is higher than those of the whole of the two urban areas.

However, land subsidence is not significant in some other areas located on carbonate rocks or soft soil area. For example, the rate of land subsidence in Daqiao carbonate rock belt is lower than −5 mm/yr, indicating that the surface is relatively stable. Therefore, an area located on carbonate rock or soft soils is not sure to subside, but an area of subsidence requires natural conditions such as the carbonate rock or soft soils. In summary, natural factors are necessary but not sufficient conditions for land subsidence.

#### 5.2.2. Human Activities

**Figure 9:**
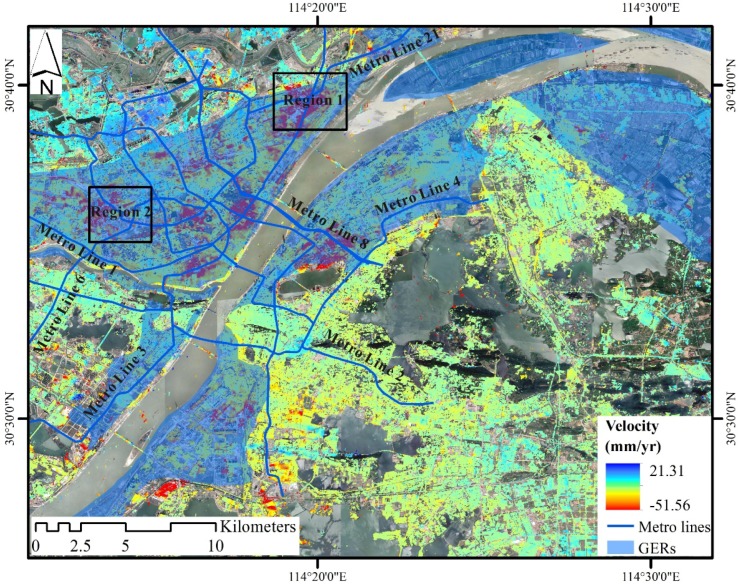
Map of the GERs and Metro Networks of Wuhan city.

According to the government’s planning for utilization of the groundwater resource, all four major areas of subsidence are located in the groundwater exploitation regions (GERs) wherein large quantities of groundwater is continuously pumped (Figure 9). Groundwater extraction will increase the fluctuation of groundwater levels. That results in the compaction of highly compressible soft soils and the dissolution of carbonate rocks or suffusion processes. Therefore, land subsidence occurs.

Many subways have been built such as Metro Lines No. 3, 6, 8 and 21, or are under construction such as Metro Lines No. 5, 7 and 11, during our study period 2015−2018. Digging subway tunnels inevitably disturb the surrounding soil, and land subsidence is more likely to follow, especially in areas of soft soil and carbonate rock. As shown in Figure 9, several centers of severe subsidence areas are distributed along the metro lines such as Region 1.

**Figure 10:**
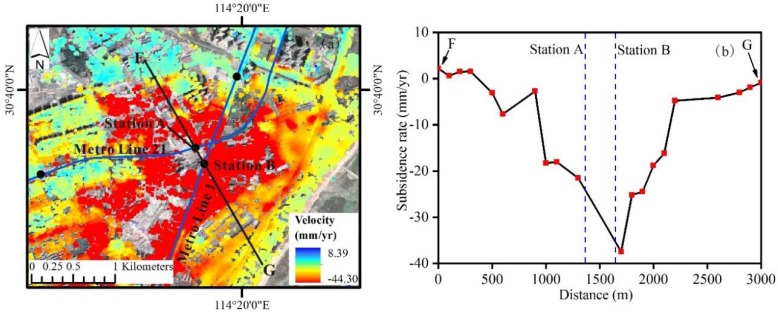
Maps show subsidence rate in Region 1 (**a**), and a subsidence profile passing through stations A and B (**b**).

In Region 1 (Figure 9), the subway lines have a high density and two metro lines intersect, namely Metro Lines No. 1 and 21, see Figure 10a. The intersection is near subway Station A and B that are situated at the center of subsidence area. The rate of subsidence reaches up to −44.30 mm/yr. A subsidence profile passing through stations A and B is shown in Figure 10b. The rate of subsidence decreases with the distance to subway stations. Therefore, subway construction can affect land subsidence.

**Figure 11:**
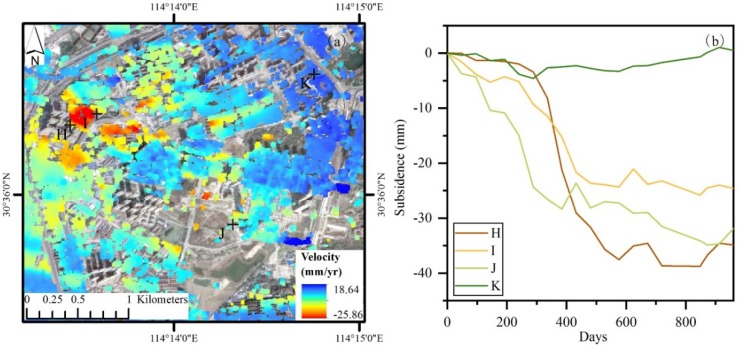
Maps show subsidence rate in Region 2 (**a**), and time-series subsidence at the four points H-K (**b**).

Wuhan city’s urban construction has entered into a stage of rapid growth during our study period 2015−2018. The annual investment in urban construction exceeds 20 billion dollars and many new buildings and transport facilities are constructed. Building a foundation often requires pumping groundwater during excavation, which could result in subsidence. In addition, when the soil underneath a building could no longer support the loading, the building will start to settle. Traffic loading also has much more influence on land subsidence because it can cause foundation deformation. Region 2 (Figure 9) is a new central business district (CBD) of the city where many high-rise buildings concentrated in, such as Wuhan Center Tower (438 m). Many new buildings and transport facilities have been constructed or being constructed. The rate of subsidence is shown in Figure 11 and severe subsidence are detected. Four typical PS points (i.e., H, I, J, and K) are selected to analyze the subsidence (Figure 11).

**Figure 12:**
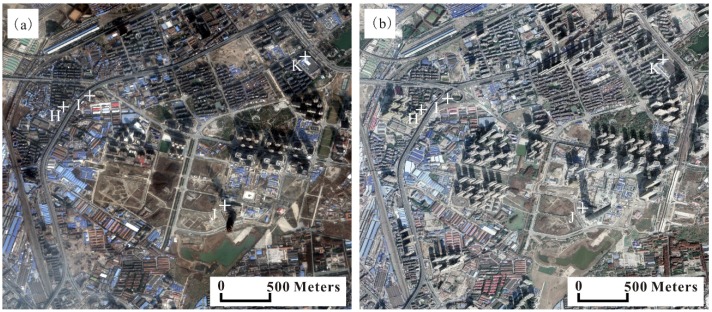
Maps show the satellite images of Region 2 on 21 January 2015 (**a**) and 9 December 2017 (**b**).

**Figure 13:**
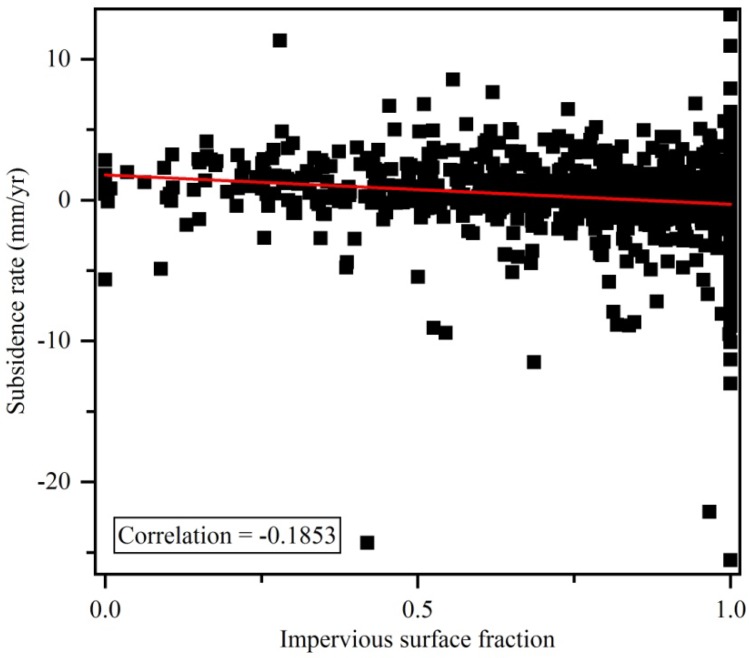
The correlation between subsidence rate and impervious surface fraction.

Points H, I and J are close to new buildings, new roads and a high-rise building, respectively (Figure 12). Point K is located on a stable surface. Points H, I and J subside greatly over time compare to point K. In addition, there is a correlation between subsidence and impervious surface fraction, see Figure 13. Thus, we can infer that urban construction such as buildings and transport facilities may drive subsidence.

In this city, soft soils or carbonate rocks are widespread, but only these areas with intensive human activities show severe subsidence, so natural conditions provide a basis for subsidence and make subsidence possible. Human activities are driving factors and make subsidence happen. Therefore, land subsidence is caused by a combination of natural conditions and human activities.

## 6. Conclusions and Future Work

Our study employs SBAS-InSAR method with Radarsat-2 data for long-term monitoring of land subsidence in a megacity, Wuhan city. The InSAR results are validated by leveling data, and the causes of subsidence are investigated. The results allowed us to draw the following conclusions: (i) SBAS-InSAR method with Radarsat-2 data could be used for longtime monitoring of land subsidence with acceptable accuracy in Wuhan city; (ii) natural conditions provide a basis for subsidence and make subsidence possible while human activities are driving factors and make subsidence happen.

Despite our success of longtime monitoring of subsidence in a megacity, Wuhan city, other advanced InSAR methods could also be investigated, such as PS-InSAR. Future study will be focused on the causes of subsidence and its spatial differences using spatial regression models. While much work has been conducted to derive land subsidence information in so many cities, the potential applications of subsidence information are rarely discussed. It is also important to explore the application of subsidence information to disaster prevention, urban planning and hydrological modeling.

## Figures and Tables

**Table 1:** Parameters of Radarsat-2 WUF SLC images.

Parameters	Description
Product type	Radarsat-2 WUF SLC
Track no.	226
Band	C
Wavelength (cm)	5.5
Revisit frequency (day)	24
Incidence angle (degree)	30–50
Range resolution (m)	1.6
Azimuth resolution (m)	2.8
Orbit direction	Descending

**Table 2:** Summary of the previous studies of land subsidence in Wuhan city.

Previous Studies	Data	Method	Subsidence Rate	Reference
Zhou et al.	15 C-band Sentinel-1A images, interferometric wide TOPS acquisition mode, VV polarization, ascending orbit, covering most of Wuhan city	SBAS-InSAR	−82–18 mm/yr	[5]
Bai et al.	12 X-band TerraSAR-X images, stripmap acquisition mode, HH polarization, ascending orbit, covering major urban areas of Wuhan city	PS-InSAR	−63.7–17.5 mm/yr	[41]
Costantini et al.	45 X-band COSMO-SkyMed images, stripmap acquisition mode, HH polarization, covering most of HK	PS Pair InSAR	−80–40 mm/yr	[42]
Benattou et al.	36 C-band Sentinel-1A images, interferometric wide TOPS acquisition mode, VV polarization, ascending orbit, covering major urban areas of Wuhan city	PS-InSAR	−127–23 mm/yr	[53]
